# Real-time prediction of formation pressure gradient while drilling

**DOI:** 10.1038/s41598-022-15493-z

**Published:** 2022-07-05

**Authors:** Ahmed Abdelaal, Salaheldin Elkatatny, Abdulazeez Abdulraheem

**Affiliations:** College of Petroleum Engineering and Geosciences, King Fahd University of Petroleum and Minerals, Dhahran, 31261 Saudi Arabia

**Keywords:** Geology, Geophysics, Engineering

## Abstract

Accurate real-time pore pressure prediction is crucial especially in drilling operations technically and economically. Its prediction will save costs, time and even the right decisions can be taken before problems occur. The available correlations for pore pressure prediction depend on logging data, formation characteristics, and combination of logging and drilling parameters. The objective of this work is to apply artificial neural networks (ANN) and adaptive neuro-fuzzy inference system (ANFIS) to introduce two models to estimate the formation pressure gradient in real-time through the available drilling data. The used parameters include rate of penetration (ROP), mud flow rate (Q), standpipe pressure (SPP), and rotary speed (RS). A data set obtained from some vertical wells was utilized to develop the predictive model. A different set of data was utilized for validating the proposed artificial intelligence (AI) models. Both models forecasted the output with a good correlation coefficient (R) for training and testing. Moreover, the average absolute percentage error (AAPE) did not exceed 2.1%. For validation stage, the developed models estimated the pressure gradient with a good accuracy. This study proves the reliability of the proposed models to estimate the pressure gradient while drilling using drilling data. Moreover, an ANN-based correlation is provided and can be directly used by introducing the optimized weights and biases, whenever the drilling parameters are available, instead of running the ANN model.

## Introduction

Formation pressure is exerted by the fluids within the rock pore space. At certain depth, the normal gradient originates from the saltwater column weight extended from the surface to the point of interest. The deviation from the normal trend can be described as abnormal which can be either subnormal or overpressure^1^. Normal pressure is not constant, and it depends on the amounts of dissolved salts, fluid types, gas presences and temperature gradient. Supernormal or overpressure is the formation pressure exceeding the normal hydrostatic pressure while subnormal pressure is the one that is lower than the normal pressure. Supernormal is created by normal pressure in addition to an extra pressure source. The excess pressure may be attributed to different reasons which may be geological, mechanical, geochemical and combined^2^. Abnormal pressure zones may lead to severe technical and economic issues such as kicks and blowouts. Subnormal pressure may lead to loss of circulation and differential pipe sticking resulting in setting additional casing strings (higher drilling costs)^2^. Accurate real-time formation pressure estimation may provide enhanced well path and casing design, better wellbore stability analysis, effective mud program and reduced overall drilling costs^3,4^.

Formation pressure estimation can be either quantitative or qualitative. Most of these techniques depend on comparing the normal trend lines with the observed ones graphically to pick the anomalous changes that may refer to abnormal pressure zones. The existing techniques in the literature utilized well logs, strata properties and drilling parameters. Hottman and Johnson^5^ were the first to estimate the pore pressure based on shale logging data by constructing cross plots that relate the pressure gradient to resistivity ratio or sonic travel time difference between the observed and the normal trend. Matthews and Kelly^6^ utilized a semi-log scale for Hottman and Johnson correlation. Pennebaker^7^ replaced the sonic travel time difference utilized by Hottman and Johnson^5^ with the sonic travel time ratio. The author estimated the pore pressure from an X–Y cross plot like the one belongs to Hottman and Johnson. This technique used a single trendline for a certain rock type globally, but this may not be true for all rock types. Eaton^8^ confirmed that formation pressure and overburden pressure gradients affect log-derived properties. As a result, the Hottman and Johnson correlations should be expanded to include overburden stress effect. Eaton^8^ proposed an empirical model based on sonic data to predict the pressure gradient in shale formations.

Gardner et al.^9^ analysed the data used by Hottman and Johnson and introduced another way to estimate the formation pressure by involving the overburden pressure. Bowers^10^ mentioned that a power relationship exists between effective stress and sonic velocity. The author estimated the formation pressure using sonic data after rearranging the power equation and replacing the effective stress with $\left({\alpha }_{V}-pore pressure\right)$. Shell introduced another sonic-based prediction technique called Tau model by introducing a “Tau” parameter in the equation of the effective stress^11,12^. Foster and Whalen^13^ were the first to use the equivalent depth method, a vertical method, to estimate the formation pressure from electrical logging. Moreover, Ham^14^ utilized the equivalent depth approach with sonic, resistivity and density to predict the formation pressure and drilling fluid weight needed in Gulf Coast wells. Eaton^15,16^ introduced empirical models based on resistivity or conductivity to estimate the pressure gradient in shale using well logging. This method can be fairly used in the sedimentary basins where under-compaction is the main source of overpressure^17,18^. Based on the drawbacks of the solo usage of ROP as an indicator of pore pressure, ROP should be corrected or normalized to consider the variation in different drilling parameters. Bingham^19^ proposed the D exponent as an attempt to correct the ROP for the variations in weight on bit (WOB), RS and well diameter. Jorden and Shirley^20^ proposed a modification to Bingham approach by introducing another term called d_exp_. Rehm and McClendon^21^ adjusted Jorden and Shirley d_exp_ by including the effect of drilling fluid density change. Quantitatively, formation pressure can be estimated using d_c_ values by Eaton method and ratio method. Eaton^15^ and Contreras et al.^22^ observed that the corrected d_exp_ graph is very analogous to the resistivity graph. Therefore, Eaton developed a prediction model for formation pressure gradient using estimated dc, normal d_c_ value, and the gradients of overburden and normal formation pressures. The ratio method was proposed as a simple technique to estimate the pore pressure from d exponent or resistivity or sonic data without overburden pressure^1^.

### Artificial intelligence

AI is an engineering science that uses high computational capabilities to develop computer programs to solve problems by mimicking human brain intelligence^23,24^. AI has different techniques such as ANN, ANFIS, functional networks, and support vector machine that show robust performance and high accuracy for classification and prediction^25^. AI is extensively utilized in different branches of engineering, medicine, economics, and military^26^. AI has been broadly applied in oil and gas industry because it has not only the capability to solve complicated issues, but it also represents them with a high accuracy^27^. Intelligent models were developed for various targets such as estimating the equivalent circulation density in real-time^28–30^, pore pressure estimation while drilling^31,32^, porosity prediction^33^, resistivity prediction^34^, predicting mud rheological properties^35–39^, predicting the unconfined compressive strength^40^, estimating the oil recovery factor^41^, bulk density log prediction^42,43^, well planning^44^, lithology classification^45^, fracture density estimation^46^, estimating the static elastic moduli^47,48^, Poisson’s ratio prediction^49–51^, and prediction of formation tops^52^.

### AI-based formation pressure prediction

Few studies applied different AI techniques to estimate the formation pressure. Li et al.^53^ utilized ANN to estimate the formation pressure in the Saertu and Xingshugang oil fields in Daqing. The authors included input parameters like sonic transit time, gamma ray (GR), natural potential, and pipe pressure. Hu et al.^54^ employed ANN to estimate the pore pressure. The authors included inputs such as depth, density, sonic transit time, and GR. Keshavarzi and Jahanbakhshi^55^ applied neural networks to estimate the gradient in Asmari field. The inputs included porosity, permeability, density, and depth. Aliouane et al.^56^ introduced ANN model to estimate the formation pressure from well logs in shale gas reservoir. Rashidi and Asadi^57^ proposed ANN model to estimate the formation pressure utilizing mechanical specific energy and drilling efficiency. Ahmed et al.^58^ utilized ANN to create a prediction model for formation pressure using seven inputs containing a combination of well logs and drilling data. Ahmed et al.^59^ compared five machine learning techniques to predict the formation pressure with the same input parameters utilized in Ahmed et al.^58^ work.

The provided models in the literature used some logging data, which may not be available while drilling as logging while drilling (LWD) is not used in all wells. Even if the LWD is present in the drill string, it is placed tens of feet above the bit that does not reflect the instantaneous response of the formations being penetrated in real-time. Other models used some reservoir properties derived from either logging data or lab measurements that limit their usage while drilling. The motivation is to develop a way to forecast the formation pressure gradient in real-time while drilling by using available drilling data only without combining them with other data that are not available in all wells. By doing so, we are maximizing the benefits of the available drilling data without involving higher costs to predict a crucial parameter that enhances the drilling operations technically and economically. The goal of this study is to use ANNs and ANFIS to propose two models for formation pressure gradient prediction in real-time using the available drilling data without additional costs. Moreover, an ANN based correlation is provided to use it directly to estimate the gradient. Unlike the developed empirical equations, the models in this study do not need a normal pressure trend to estimate the gradient.

## Methodology 

**Figure 1:**
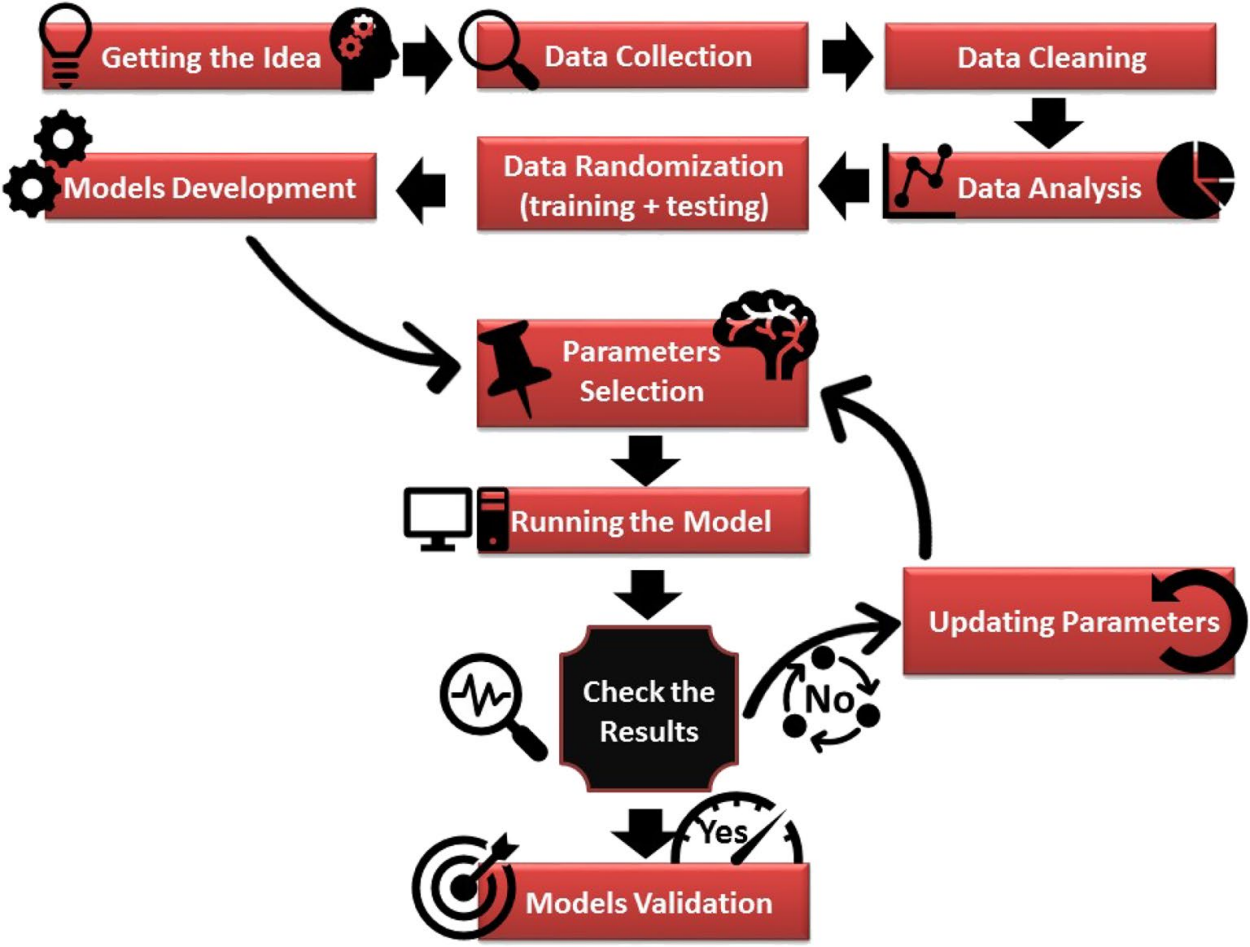
Flow chart of the methodology conducted in the study.

The methodology started with data collection followed by data cleaning and filtration. Then, data analysis was performed to get more insights about the data sets. After that, data were randomly divided while ensuring that the data sets are representative. The next stage was to select initial model parameters for the first runs. The parameters were updated, and the process was repeated until getting the best results. Once the optimum results came out, the model hyperparameters were extracted. Finally, the models were validated by blind holdout dataset that was not involved in developing the predictive models. Figure 1 briefly shows the methodology conducted in this work to develop the AI models.

## Data processing and analysis 

### Data description

**Figure 2:**
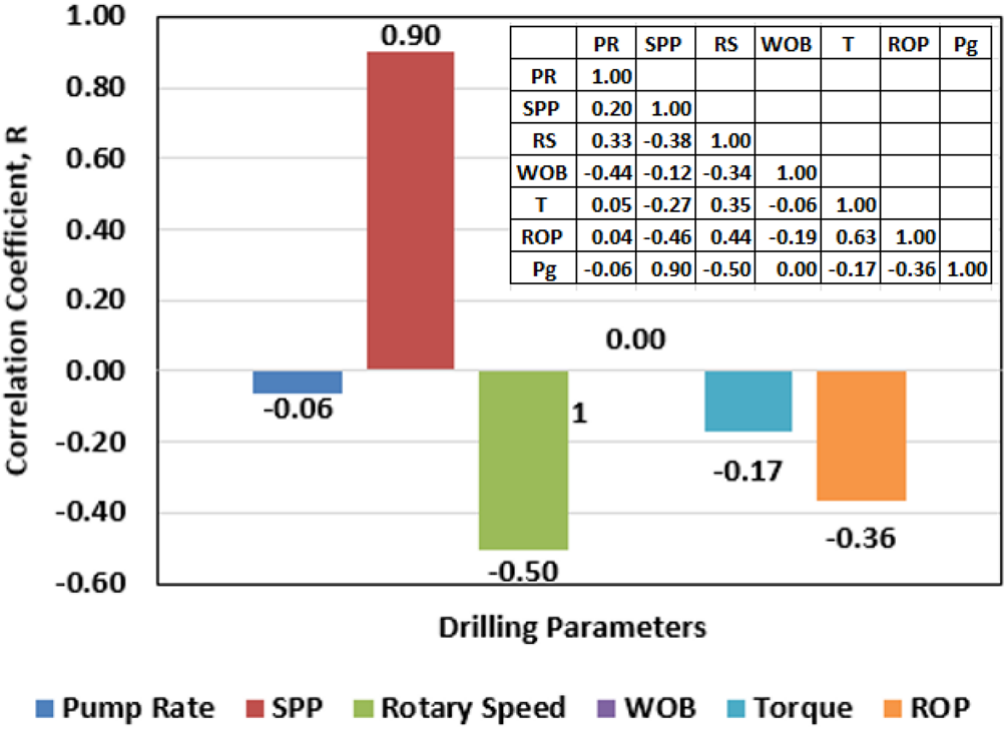
R-values between each input and the formation pressure gradient along with a table containing the R-values between each two variables.

**Figure 3:**
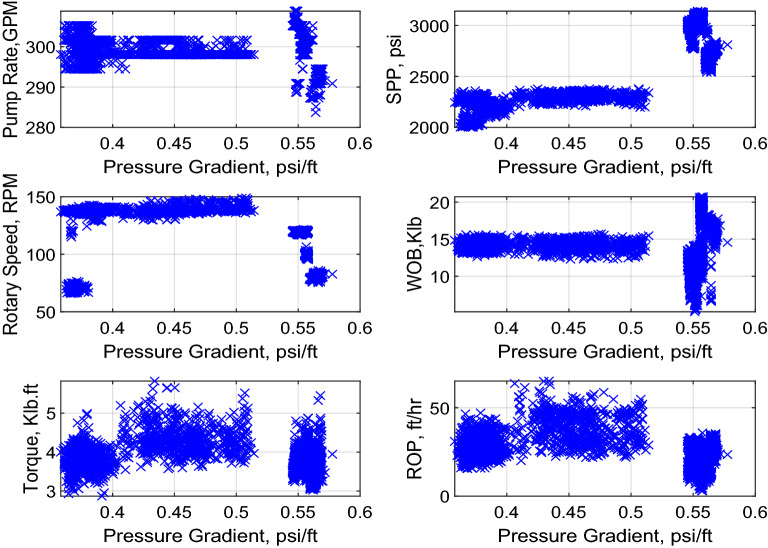
Cross-plots of pressure gradient versus different drilling parameters.

A set of data containing around 3145 points was provided from vertical sections in the same area. The set of data included the drilling data, the formation pressure and depth. The drilling data were utilized as inputs to feed the model to predict the formation pressure gradient as an output. These drilling data included hydraulic data like Q, and SPP, and mechanical data such as: RS, ROP, torque (T), and WOB. These drilling data can be recorded either at surface or downhole while drilling and are influenced by strata being penetrated and their fluid content. Statistical analysis was performed on the field data, and it showed that the data covered a broad range of the inputs and the output as presented in Table 1. For instance, the data had a good representation of the formation pressure gradient as it covers subnormal, normal and supernormal gradient values. Table 2 shows a sample of the field data utilized in this study. The relationship between each variable and the other variables was tested in terms of R as shown in Fig. 2. Moreover, cross-plots of each drilling parameter with pore pressure gradient were constructed as shown in Fig. 3.

**Table 1:** Data statistical Analysis.

Statistical Parameter	Q (gal/min)	SPP (psi)	RS (rpm)	WOB (klb)	T (klb.ft)	ROP (ft/h)	Pressure gradient (psi/ft)
Minimum	283.69	2000.54	65.92	5.21	2.87	3.02	0.36
Maximum	308.83	3140.57	148.96	20.73	5.82	65.08	0.58
Mean	299.52	2599.73	118.73	14.13	3.85	27.22	0.48
Standard deviation	4.53	377.66	22.05	2.38	0.38	9.43	0.08
Skewness	−0.78	0.09	−0.86	−0.10	0.99	0.45	− 0.36
Kurtosis	3.55	1.28	2.47	4.44	4.84	3.60	1.36

**Table 2:** A sample of the field data utilized to build the models.

Pump rate (PR) (gal/min)	SPP (psi)	RS (rpm)	ROP (ft/h)	Pressure gradient (psi/ft)
301.6	2049	70.04	19.27	0.376
301.6	2222	138.23	34.31	0.388
298.0	2271	139.52	24.51	0.477
298.0	2304	141.19	44.61	0.462
298.0	2333	145.67	47.19	0.489
290.8	2778	80.44	29.91	0.568

### Data processing 

In AI, the quality of data is as significant as the prediction quality. As a result, the data set was cleaned by eliminating the unrepresentative values such as −999 values, and NAN (not a number). Then, the outliers which are the observations located outside the overall pattern of a distribution should be removed because they may cause serious problems in statistical analysis^60^. Outliers may exist owing to human and/or instrument error. Outlier detection can be conducted by many ways such as Z-Score (removing values located away from the mean by more than a certain number of standard deviations) and a box-and-whisker plot (removing values located beyond the upper and the lower limits determined by dividing the data into four quartiles)^61^. The quality and the reliability of the inputs were checked by various techniques like comparing the recorded variables with the ranges of the equipment and with the similar variables in the offset wells within the field. Moreover, the output was compared to the formation pressure gradient values produced by known trends of the gradient of the strata in the selected area. The check showed a good matching between the recorded and produced pressures indicating the reliability of the measurements.

### Selection criteria of the inputs

The strata characteristics influence the drillability of the geological column because the properties control the impedance to drill through strata. The drilling data may by some means mirror the resistance faced while drilling different formations. Rotary speed and weight on bit can be adjusted based on the nature of the formations^62^. Additionally, the generated cuttings during drilling have impacts on the pressures and rates of the pump required to ensure good hole cleaning. All the previous drilling parameters and the formation type play an important role in controlling penetration rate^63,64^. Consequently, the drilling data can somehow reflect the drilled formations nature, and in turn, their formation pressures. ROP can be used as an indicator to identify supernormal layers while drilling. ROP was included to develop these models since it includes the effect of other drilling variables like WOB. Furthermore, RS was utilized to build the models since it indirectly contains the effect of the T. For the simplicity of the model, two mechanical variables (ROP and RS) were employed along with two hydraulic variables (SPP and Q).

## Development of the pore pressure gradient models 

### ANN model 

**Figure 4:**
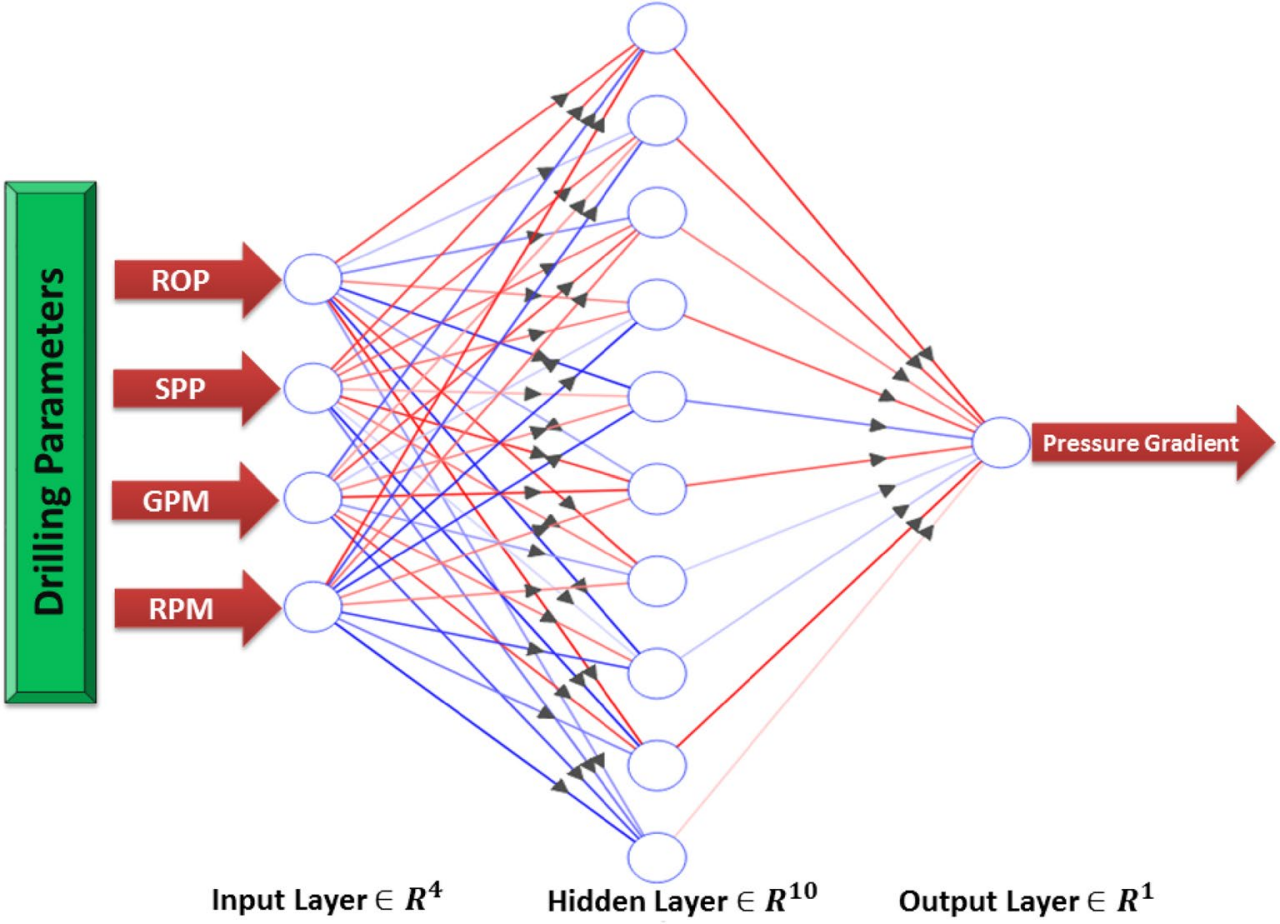
Schematic of the structure of the developed ANN.

After checking the quality of the selected dataset. The obtained data had been divided into two groups with 3:1 ratio for training and testing. The ANN model hyperparameters, including different combinations of various available options for ANN hyperparameters, were optimized by testing many scenarios per each parameter. The different options for each ANN parameter and the optimum options are listed in Table 3. The R, coefficient of determination (R^2^) and AAPE were computed by Eqs. (1), (2) and (3) as presented in Supplementary Appendix 1. The hyperparameters providing the highest R, R^2^ and the minimum error (RMSE, MSE and AAPE) had been selected. It was found that the optimum number of neurons is 10 occupying only one hidden layer. The model was built using newcf network with Levenberg–Marquardt algorithm (trainlm) as a training function to obtain the optimum weights and biases using 0.12 learning rate. Log-sigmoidal-type (logsig) activation function was used as a transfer function connecting the input and the hidden layer and a linear-type (purelin) activation function linked the hidden and output layers. Figure 4 shows a typical structure of the proposed ANN model.

**Table 3:** Parameters optimization process.

Parameter	Options/range	Optimum option
Hidden layers number	1 to 4	1
Neurons number per each layer	1 to 40	10
Learning rate	0.01 to 0.9	0.12
Network	fitnet–newfit–newlm–newff–newpr–newfftd–newdtdnn–newelm–newnarx–newcf	(newcf)
Training function	trainlm–trainbr–traincgb–traincg–trainrp–trainb–trainbr–trainbfg–traincgf–traincgp–traingdx–trainoss–trainr–trainscg	(trainlm)
Transfer function	logsig–satlin–softmax–hardlim–purelin–compet–hardlims–poslin–satlins–radbas–tansig–tribas	(logsig)

**Figure 5:**
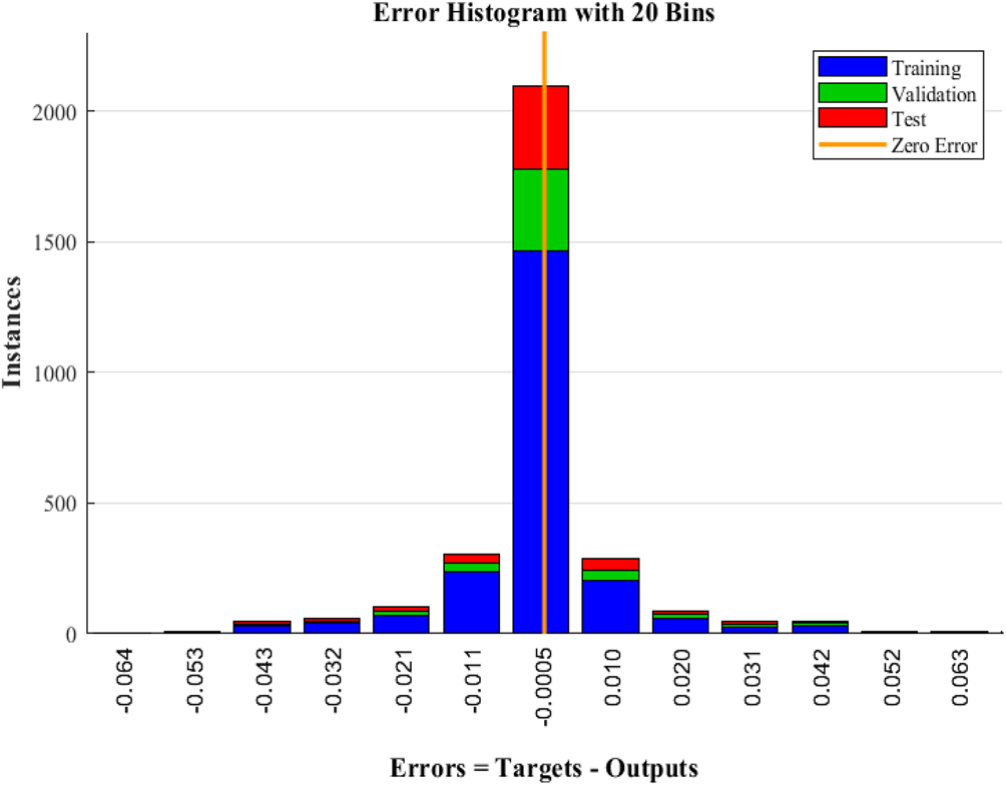
Error histogram of the developed ANN model.

**Figure 6:**
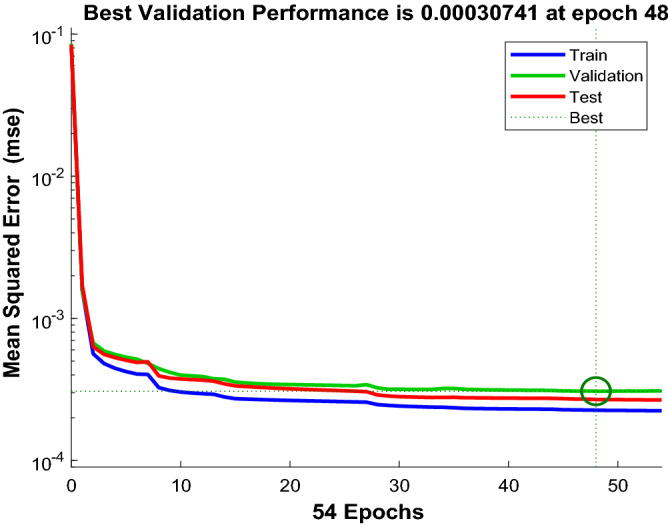
Training performance in terms of MSE showing the best validation at epoch 48.

**Figure 7:**
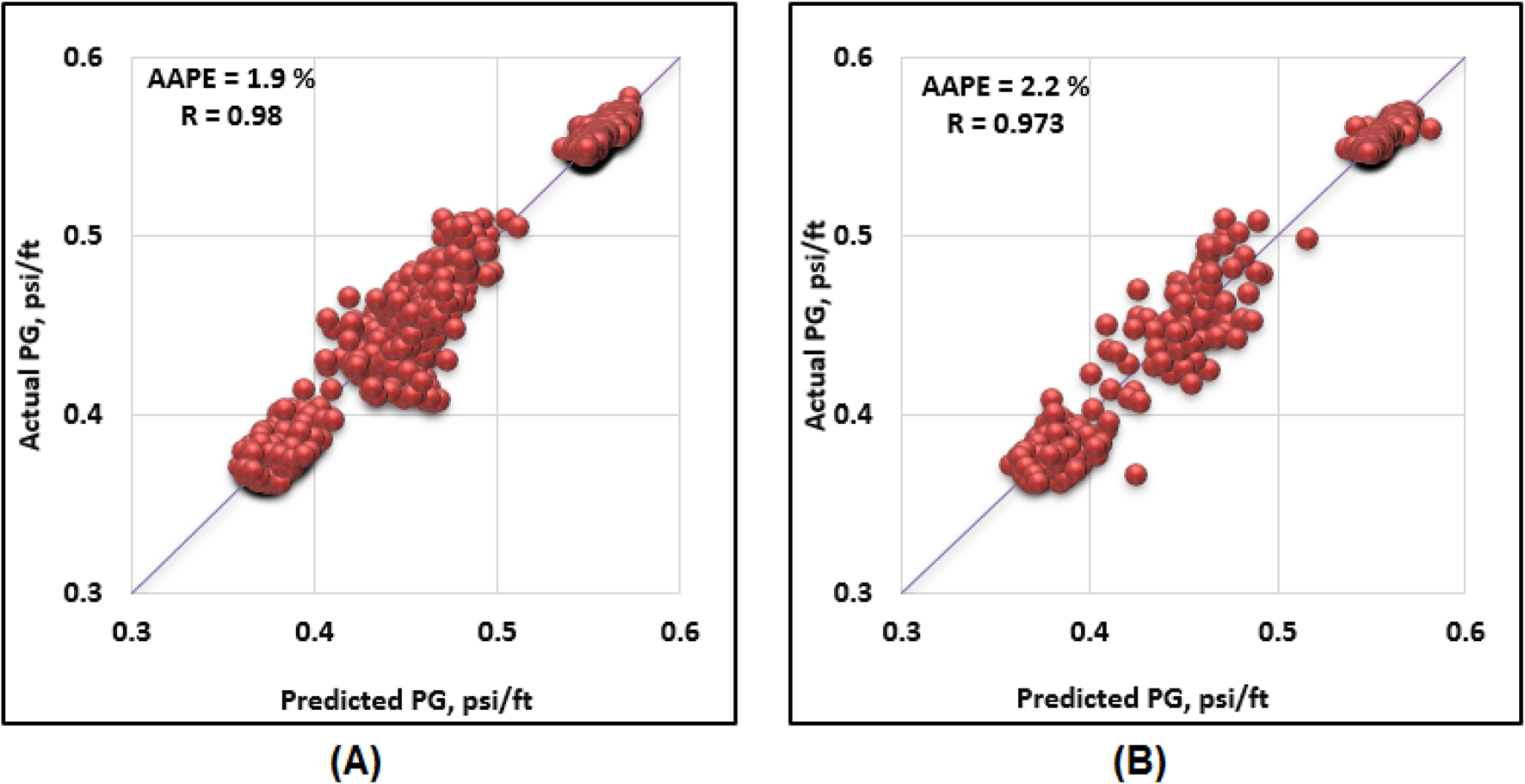
Cross-plots of the estimated versus recorded target values (**A**) training, and (**B**) testing (ANN model).

**Figure 8:**
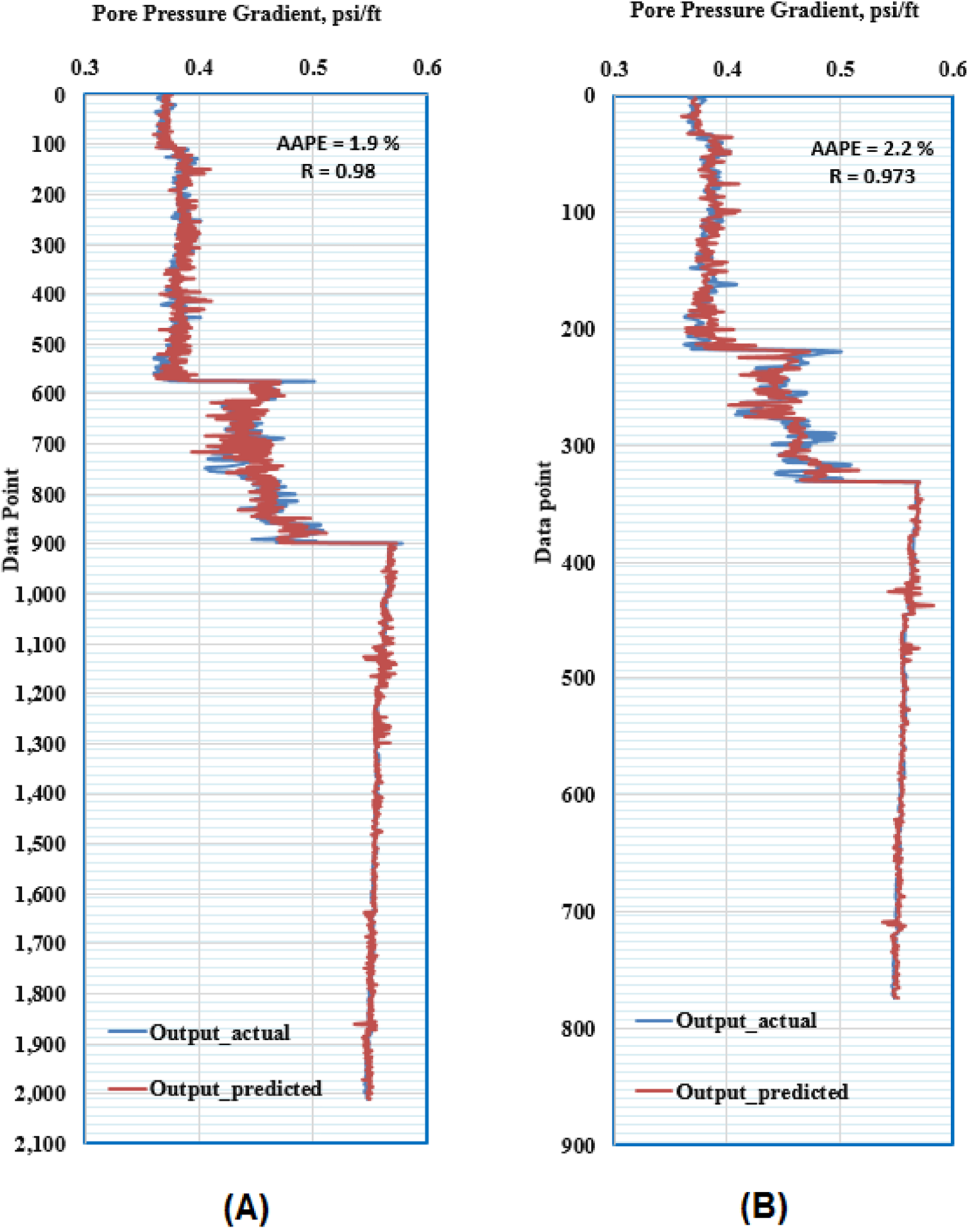
Formation pressure gradient profiles (**A**) training, and (**B**) testing (ANN model).

The proposed ANN model consists of three layers. The first layer contains the inputs; the second layer contains the neurons with their weights and biases and the third layer is the output layer. The input parameters for the model were Q, ROP, SPP and RS. The ANN model predicted the formation pressure gradient with high R of 0.981 and 0.973 for training and testing respectively. Moreover, the RMSE ranges between 0.015 to 0.018 and AAPE does not exceed 2.22% for training and testing. The obtained results for training and testing are summarized in Table 4. The error (predicted—actual) histogram shows that most predicted values have very small error ranging between − 0.02 to 0.02 psi/ft as shown in Fig. 5. The network training performance was monitored against mean squared error as shown in Fig. 6 with the best validation at epoch 48. Figure 7 presents the cross plots of the estimated versus the recorded target values showing the points coinciding with the 45° line. The recorded and predicted target values were graphed on the same plot to observe the variations through the chosen intervals, as presented in Fig. 8, indicating high estimation accuracy.

**Table 4:** Training and testing results for ANN model.

Parameter	Training	Testing
R	0.98	0.97
R^2^	0.96	0.95
AAPE (%)	1.90	2.21
RMSE (psi)	0.015	0.018

#### New empirical correlation for formation pressure gradient

The weights and biases were extracted from the optimized ANN model as listed in in Table 5 to provide an empirical equation for predicting the pore pressure gradient from the available drilling parameters. The developed equation in the normalized form is given by Eq. (1) and may be utilized after normalization stage of the input parameters to be in the range of −1 to 1 as given by Eq. (2).

1 $${Pg}_{n}=\left[\sum_{i=1}^{N} {w}_{{2}_{i}}\left(\frac{1}{1+exp\left(-\left({PR}_{n}*{w}_{{1}_{i,1}}+{SPP}_{n}*{w}_{{1}_{\mathrm{1,2}}}+{RS}_{n}+{w}_{{1}_{i,3}}+{ROP}_{n}*{w}_{{1}_{i,4}}+{b}_{1,i}\right)\right)}\right)\right]+{b}_{2}$$

where ${Pg}_{n}$ is the normalized $Pg$*,* $N$ is the neurons number, i.e. 10, ${w}_{{1}_{i}}$ is the weight associated with each feature between the input and the hidden layer, ${w}_{{2}_{i}}$ is the weight associated with each feature between the hidden and the output layer, ${b}_{{1}_{i}}$ is the bias attached to each neuron in the hidden layer, ${b}_{2}$ is bias of the output layer.

2 $${Y}_{{i}_{nor}}=2\left(\frac{{Y}_{i}- {Y}_{i min }}{{Y}_{i max}- {Y}_{i min}}\right)-1$$

where, ${Y}_{{i}_{nor}}$ is the normalized value of variable $Y$, ${Y}_{\mathrm{i}}$ is the value of variable $Y$ at point i, ${Y}_{i min}$ is the minimum value of variable $Y$, ${Y}_{i max}$ is the maximum value of variable $Y$. The minimum and maximum values for each parameter that were used in data normalization are shown in Table 6.

**Table 5:** Extracted weights and biases for the empirical correlation.

Neuron index (i)	${w}_{1}$				${w}_{2}$	*b*_*1*_	*b*_*2*_
	${w}_{{1}_{i,1}}$	${w}_{{1}_{i,2}}$	${w}_{{1}_{i,3}}$	${w}_{{1}_{i,4}}$			
1	2.5805	−0.1332	−1.5521	2.4388	1.8362	−1.6164	3.5914
2	1.1140	0.3965	−2.0108	2.0790	−1.6815	−2.5735	
3	5.8387	31.5162	5.8181	6.0894	12.2992	0.9777	
4	6.6950	1.9846	6.4276	2.3458	−3.4529	0.4803	
5	0.8564	1.9802	−0.1041	2.1931	−1.7299	3.2192	
6	−6.2878	−4.4130	4.7209	1.6685	−6.8253	5.0057	
7	7.2797	15.3465	− 0.6331	−1.6916	4.0337	−2.1368	
8	−3.2785	−10.7832	1.4624	0.7178	−3.0489	−3.1494	
9	−6.7507	−4.1005	2.3319	1.9685	−4.9894	−4.8898	
10	−1.6419	−1.7707	−0.8873	−2.5208	−2.2377	−3.4025

**Table 6:** Values used for data normalization.

Statistical parameter	Q (gal/min)	SPP (psi)	RS (rpm)	ROP (ft/h)	Pg (psi/ft)
Minimum	283.69	2000.54	65.92	3.02	0.36
Maximum	308.83	3140.57	148.96	65.08	0.58

#### Steps to estimate the pressure gradient using the ANN-based correlation

1. Normalize the input drilling parameters into ${\mathrm{PR}}_{\mathrm{n}}, {\mathrm{SPP}}_{\mathrm{n}}, {\mathrm{RS}}_{\mathrm{n}} {\mathrm{and ROP}}_{\mathrm{n}}$ using Eq. (2) and statistical data in Table 6.
2. Calculate the normalized value of the output ${Pg}_{n}$ using Eq. (1) and the optimum weights and biases listed in Table 5. The input data should be ordered as follows: pump rate (GPM), SPP (psi), rotary speed (RPM) and ROP (ft/h), with the same units.
3. The obtained ${Pg}_{n}$ is denormalized to an actual $\mathrm{Pg}$ value by Eq. (3):
  3 $$Pg={0.11 (Pg}_{n}+1)+0.36$$
  where, ${\mathrm{Pg}}_{\mathrm{n}}$ is the normalized $\mathrm{Pg}$ estimated by the developed correlation, $\mathrm{Pg}$ is the actual value (psi/ft).

### ANFIS model

**Figure 9:**
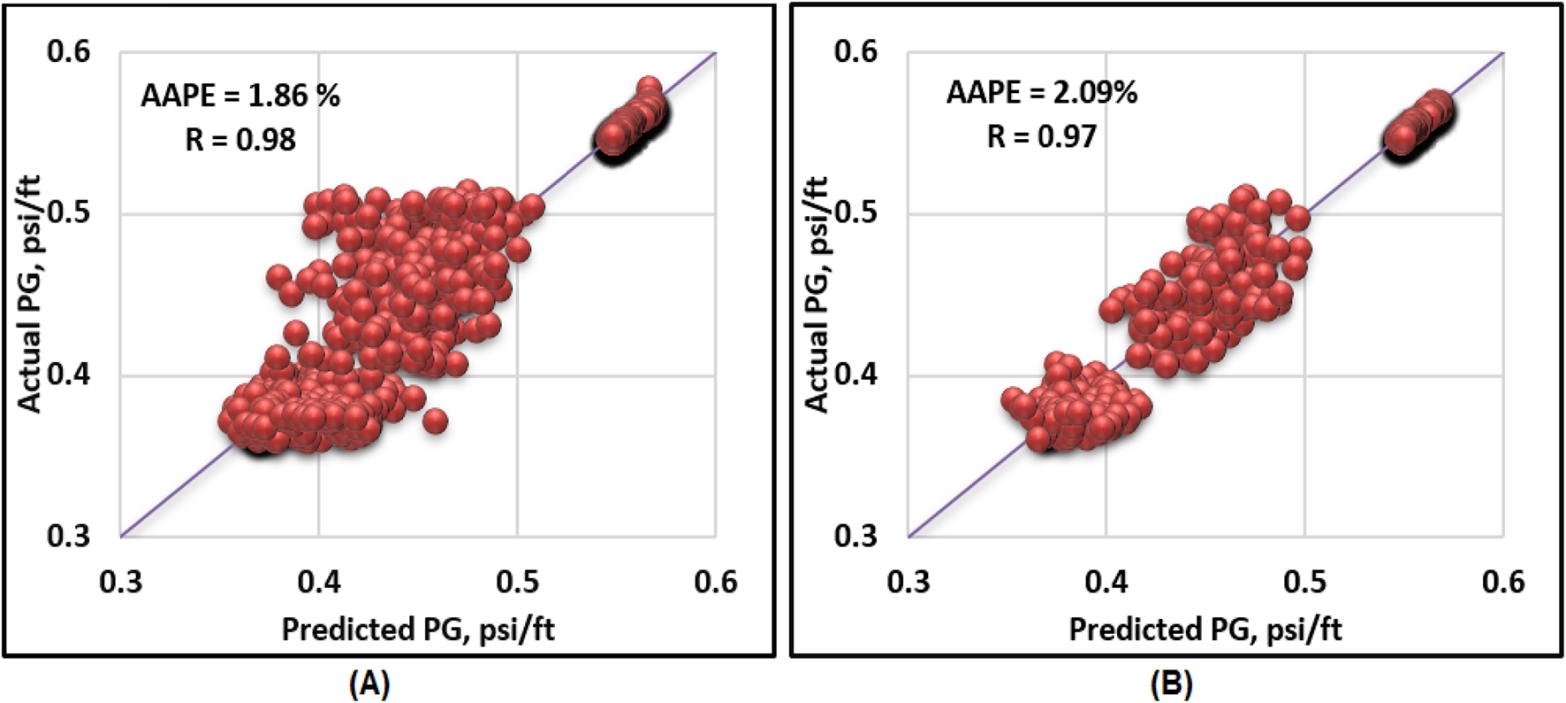
Cross-plots of the ANFIS model (**A**) training, and (**B**) testing.

**Figure 10:**
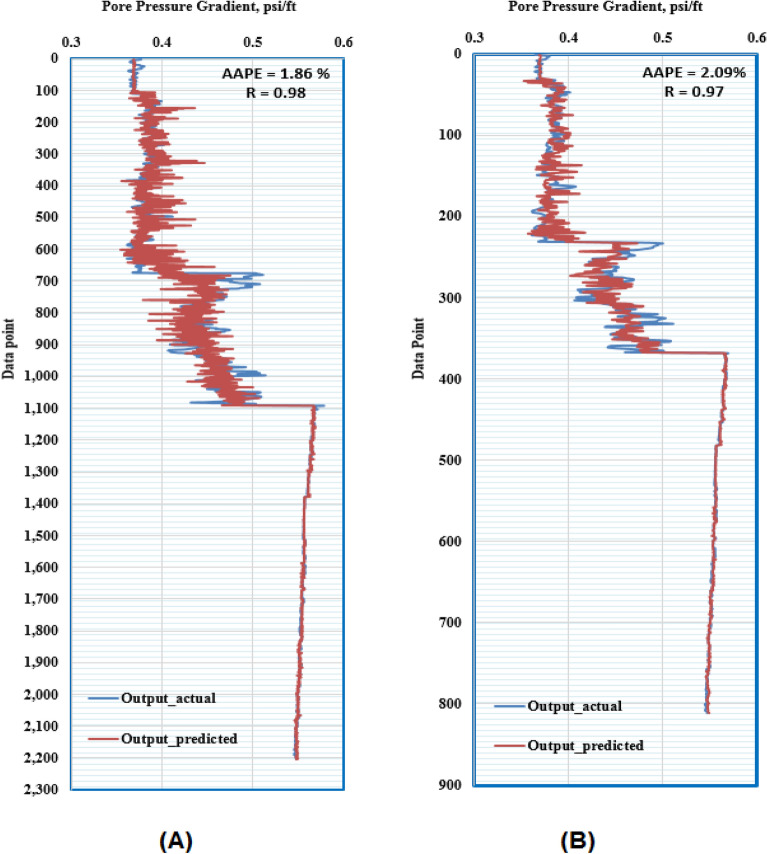
Formation pressure gradient profiles (**A**) training, and (**B**) testing (ANFIS model).

ANFIS application in petroleum engineering showed a high reliability as a predictive tool^65^. Genfis 1 that uses grid partitioning and Genfis 2 that uses subtractive clustering were both tested to obtain the model. Genfis 2 provided better results compared to Genfis 1 consequently, the ANFIS model was created by the subtractive clustering technique. The optimization process included using different combinations of cluster radius size and number of iterations. The model was built using the Sugeno–Fis type with a cluster radius of 0.2 and 400 iterations resulting in the best results. The ANFIS model predicted the target with high R of 0.98 and 0.97 for training and testing. Moreover, the RMSE was around 0.02 psi/ft and AAPE does not exceed 2.1% for training and testing. The obtained results for training and testing are summarized in Table 7. Figure 9 presents the cross plots of the predicted versus recorded target values showing the points coinciding with the 45° line. The recorded and estimated values were graphed on the same plot to observe the variations along the chosen intervals, as presented in Fig. 10, indicating high prediction accuracy.

**Table 7:** Training and testing results for ANFIS model.

Parameter	Training	Testing
R	0.98	0.97
R^2^	0.96	0.95
AAPE (%)	1.86	2.09
RMSE (psi)	0.016	0.018

### Models validation

**Figure 11:**
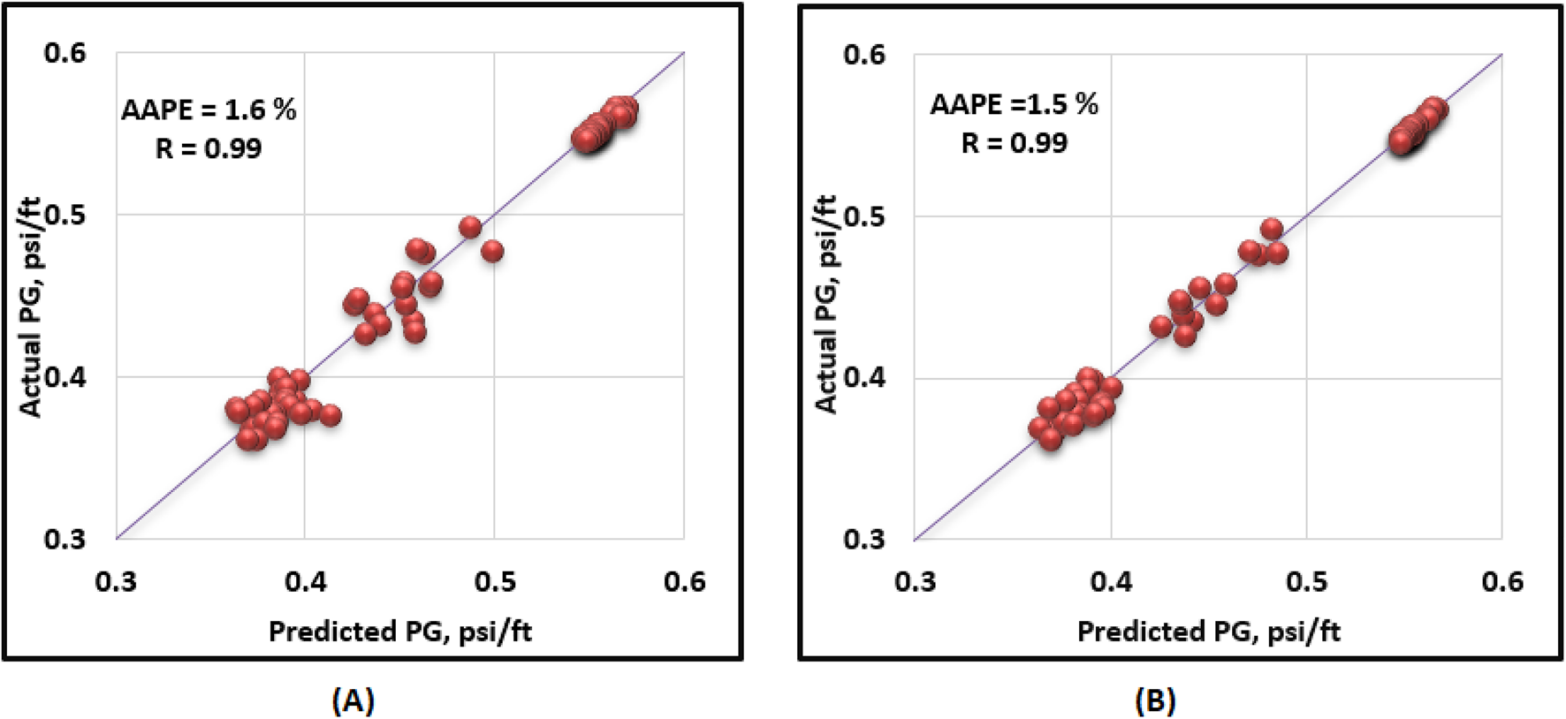
Cross-plots for validation stage (**A**) ANN, and (**B**) ANFIS.

The proposed ANN and ANFIS models were validated using a blind holdout data set that were not involved in developing the models. A data set (92 points) from the same field was collected to feed the models and compare the recorded versus the estimated pressure gradient values. The models provided continuous profiles of the target using the profiles of the drilling data. Both ANN and ANFIS predicted the target with high R of about 0.99 between the recorded and estimated target values for validation. Additionally, the RMSE was around 0.01 psi/ft and AAPE did not exceed 1.63% for the two models. Figure 11 presents the cross plots of the predicted versus recorded target values showing the points coinciding with the 45° line. The proposed models performed reasonably well when tested using testing and validation data sets that were not included in the training stage.

## Conclusion 

In this work, a novel way for estimating the formation pressure gradient using AI while drilling using the available surface drilling data was introduced. Unlike the developed empirical models in the literature, the developed models do not need a normal trend to predict the formation pressure. The developed models can be merged with any automatic drilling system to estimate the pressure gradient while drilling at low costs. Moreover, it may decrease the non-productive time by minimizing the time-consuming drilling issues by forecasting and minimizing them before they might occur. This tool may improve the drilling operations technically and economically during drilling and pre-drilling design to take the right decisions and to avoid possible issues like kick, blowout, and circulation losses. The results of this work can be listed as follows:

- The optimum parameters of the ANN model are one hidden layer containing 10 neurons, newcf network with Levenberg–Marquardt algorithm (trainlm) as a training function with 0.12 learning rate, and a log-sigmoidal as a transfer function.
- The optimum parameters of the ANFIS model based on subtractive clustering are cluster radius of 0.2, and 400 iterations.
- The proposed models can predict the pore pressure gradient with reasonable accuracy as indicated by R around 0.975, and RMSE around 0.018 psi.
- The ANN-based correlation can be directly utilized by introducing the optimum weights and biases, whenever the drilling parameters are available, instead of running the ANN model.

## Supplementary Information

Supplementary Information.

## List of symbols

*Pg*: Pressure gradient
ANN: Artificial neural network
ANFIS: Adaptive network-based fuzzy interference system
*R*: Correlation coefficient
AAPE: Average absolute percentage error
AI: Artificial intelligence
FIS: Fuzzy inference system
*R*^2^: Coefficient of determination
MSE: Mean squared error
WOB: Weight on bit
RPM: Rotating speed in revolutions per minute
ROP: Rate of penetration
GPM: Gallon per minute
SPP: Standpipe pressure
T: Torque
fitnet: Function fitting neural network
newfit: Create fitting network
newcf: Create cascade-forward backpropagation network
newelm: Create Elman backpropagation network
newlrn: Layer-recurrent network
newdtdnn: Create distributed time delay neural network
newff: Create feedforward backpropagation network
newpr: Create pattern recognition network
newfftd: Create feedforward input-delay backpropagation network
trainbr: Bayesian regularization
trainoss: One step secant backpropagation
trainlm: Levenberg–Marquardt backpropagation
trainbfg: BFGS quasi-Newton backpropagation
traingdx: Gradient descent with momentum and adaptive learning rule backpropagation
tansig: Hyperbolic tangent sigmoid transfer function
logsig: Log-sigmoid transfer function
hardlims: Hard-limit transfer function
purelin: Linear transfer function
softmax: Softmax transfer function
tribas: Triangular basis transfer function
satlin: Saturating linear transfer function
netinv: Inverse transfer function
radbas: Radial basis transfer function
b_1_: Input layer biases
b_2_: Output layer bias
*w*_*1*_: Weights linking inputs and hidden layer
*w*_*2*_: Weights linking output and hidden layer

## Subscripts

*i*: Index of each neuron in the hidden layer
*n*: Normalized value
