# Supplementary material for: Real-time prediction of formation pressure gradient while drilling
Source: Sci Rep. 2022 Jul 5;12:11318. doi: 10.1038/s41598-022-15493-z (PMC9256675; doi:10.1038/s41598-022-15493-z)
Supplement: Supplementary file 1 — Supplementary Information. [file 41598_2022_15493_MOESM1_ESM.docx]

# Appendix 1

The formula of the correlation coefficient (R), between any two variables (*x , y*), used in this study is expressed as:

$$R=\frac{k\sum xy-\left( \sum x \right)\left( \sum y \right)}{\sqrt{k\left( \sum x^{2} \right)-\left( \sum y \right)^{2}}\sqrt{k\left( \sum b^{2} \right)-\left( \sum b \right)^{2}}} (1)$$

Coefficient of determination (R^2^) is expressed as:

$$R^{2}=\left( \frac{k\sum xy-\left( \sum x \right)\left( \sum y \right)}{\sqrt{k\left( \sum x^{2} \right)-\left( \sum y \right)^{2}}\sqrt{k\left( \sum b^{2} \right)-(b)^{2}}} \right)^{2} (2)$$

Average absolute percentage error AAPE is expressed as:

$$AAPE=\frac{\sum\left| \frac{{Pg}_{\text{measured }}-{Pg}_{\text{predicted }}}{{Pg}_{\text{measured }}} \right|\times100}{n} (3)$$

where *Pg* is the pressure gradient, and *n* is the number of points.
